# The research advances of crosstalk between cancer-associated fibroblasts and tumor cells using co-culture organoids

**DOI:** 10.1038/s41419-026-08512-8

**Published:** 2026-02-26

**Authors:** Minghui Wang, Xiaodi Ding, Lin Chen, Zhichao Cui, Yiyi Ding, Yongmin Song, Wentong Li, Xumei Zhang

**Affiliations:** 1 School of Clinical Medicine, Shandong Second Medical University, Weifang, Shandong Province China; 2 Department of Pathology, Affiliated Hospital of Shandong Second Medical University, Weifang, Shandong Province China; 3 Department of Pathology, School of Basic Medicine Sciences, Shandong Second Medical University, Weifang, Shandong Province China

**Keywords:** Cancer microenvironment, Cancer models

## Abstract

Cancer-associated fibroblasts (CAFs) fundamentally impact on characteristics of tumor cells and are concerned with therapy resistance via extensive interplay with cancer cells and other stromal components. In addition, inherent plasticity and multifunctionality of CAFs enable cancer cells to cultivate them, leading to dynamic changes in the population of CAFs in a context-dependent manner. Despite CAFs have long been regarded as a key participant in cancer development and therefore an appealing therapeutic target, most clinical trials targeting CAFs end in failure, and even accelerate progression of cancers, indicating that dynamic complicated identity and function of CAFs far exceed the current view. Accordingly, analyzing the heterogeneous subgroups and different functions of CAFs in a context dependent mode is of great significance. To ascertain the functional interactions between CAFs and cancer cells, various three-dimensional co-culture models of organoid with CAFs and cancer cells from murine or human have been successfully established. In the review, we recapitulate the proposed methods for cultivating organoids consisted of tumor cells and CAFs as well as molecular mechanisms involving in regulating variety of CAF subgroups. Current strategies targeting tumor-promoting CAFs selectively are also discussed, offering perception and perspectives for scientific investigation and clinical trials concerning various methods targeting CAFs.

## Facts and questions

1. CAFs experience phenotypic and functional conversion when interplaying with cancer cells and exhibit multifaceted roles, such as enhancing cancer progression and stimulating cancer stem cell properties.
2. Strategies targeting CAFs to exploit more effective remedies, such as ECM modulation, direct elimination, interruption of CAFs-tumor crosstalk and CAF normalization, are still unsatisfactory, indicating that dynamic complexities of CAF identity and function are needed further research.
3. Tumor organoids co-cultured with CAFs recapitulate structure and function of solid tumors and could be a suitable model for future usage in clarifying crosstalk mechanism between CAFs and tumor cells or other stroma cells and screen of precision targeted drugs.

## Introduction

Tumor microenvironment (TME) has been supposed to be a momentous determinant for tumorigenesis, therapeutic response and prognosis [1], and cancer associated fibroblasts (CAFs) exhibit multifaceted roles in TME [2, 3]. Already in the earliest stage, CAFs facilitate cancer cell initiation via extracellular matrix (ECM) remodeling [4]. During tumor progression, CAFs are essential for invasion, metastatic spread, angiogenesis, drug resistance and cancer stem cell (CSC) self-renewal by establishing a dense tumor niche [5–7].

Targeting CAFs directly appears to be a potential precision intervention in clinical practice; actually, various methods targeting CAFs have displayed inconsistent findings, even leading to worse outcomes [8]. The heterogeneity of CAFs means that broad-targeting strategies may inadvertently disrupt protective stromal functions or fail to address context-specific interactions [9]. Moreover, uncharacterized interaction between tumor cell-derived signaling and CAFs differentiation restricts targeted inhibitor development.

In recent years, organoids have emerged as new 3D in vitro disease models that possess the advantages of perpetuating heterogeneity and genetic changes of primary tumors [10, 11]. Given the promising potential of organoids in revealing the crosstalk between tumor cells and CAFs, here, we provide a comprehensive review of this subject to provide a reference for research and practical applications of organoids to perceive the interplays and treatment response.

## Superiority of tumor organoids in cancer research

The intention of CAFs research rests with sorting out the interactions between CAFs and cancer cells using a variety of methods. Organoids offer unprecedented advantages for modeling human biology and diseases by recapitulating tissue architecture, cell-cell interactions and functional complexity [12, 13]. Key advantages of organoids include rapid scalability and ethical benefits by reducing reliance on animal experiments and high throughput cultivation; moreover, precise analysis of organoids is extremely craved to expand exertion in personalized drug screening [14].

## Organoid models with CAFs and tumor cells

CAFs are mightily heterogeneous, plastic and versatile cells with subtypes identifiable through distinct gene expression profiles and functional properties which have been highlighted by mounting single-cell RNA sequencing data [15, 16]. CAFs are actively engaged in cancer development via sophisticated interplay with tumor cells and other stroma cells in TME. Tumor organoids derived from patients share similar functions and morphological features with primary tumor and can replicate the response to chemotherapy and radiotherapy of an individual cancer patient [17, 18] (Table 1).

**Table 1:** The advantages and disadvantages of different organoid models.

Mixed organoid models	Advantages	Disadvantages
Conventional culture of tumor organoids	Easy to operate, low cost	Heterogeneous source of matrigel [31, 32], batch-to-batch inconsistencies in matrigel [31], difficulties in large-scale culture, lacking spatial control over stromal-tumor interaction [34]
Inverted suspension	Reducing contamination risks	Low throughput and inconsistent spheroid sizes [33]
3D microfluidic co-culture system	Recapitulating tumor-stromal crosstalk, imaging-based quantification [34], controlled size, large-scale organoid culture [35, 36]	Increased demand of matrigel and cells; exacerbated cell loss and complicated perfusion dynamics [39], complex and expensive devices
Acoustic droplet printing (ADP)	Precise spatial patterning of multiple cells [17, 40], faster establishment of size-controlled organoids, applicable for rare samples [41], mimicking ECM features accurately [45, 46]	Challenges in fabricating systems, bioinks short of optimal mechanical properties and biocompatibility [43], difficult to adjust balance between viscosity and cell adhesion [44], shear stress compromising cell viability [106]
3D mechano-mimetic scaffold	CAFs-derived ECM enhances cell adhesion and viability [47], native cues of decellularized ECM scaffolds [48]	Difficult to recapitulate native tissue complexity, scaffold rigidity deviating from physiological mechanical cues [50]

### Conventional culture of tumor organoids

**Fig. 1:**
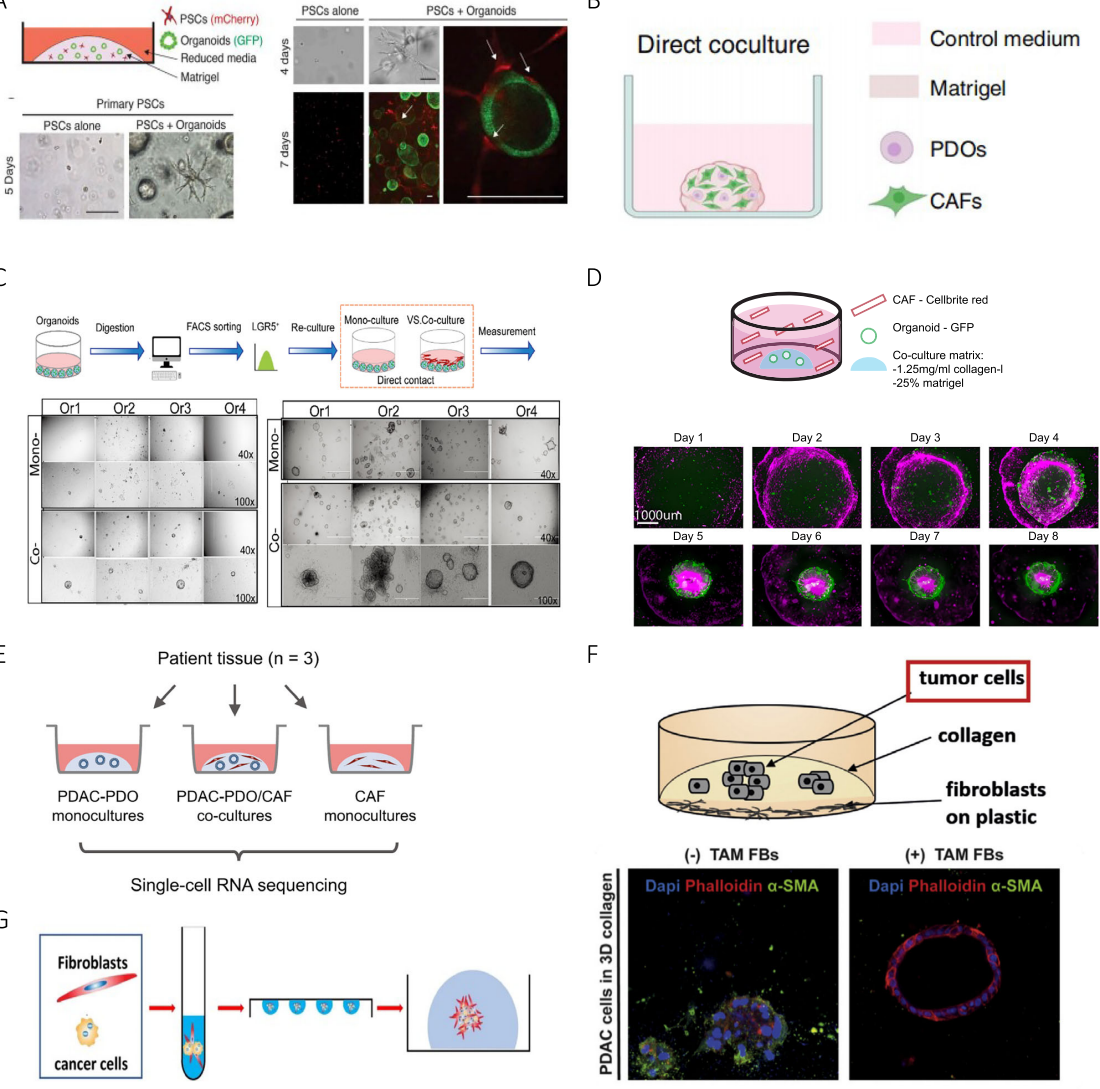
Traditional organoid co-culture models of CAFs and cancer cells. **A** Establishment of co-culture organoids composed of mouse PSCs and pancreatic cancer cells. Credit: Öhlund et al., Distinct populations of inflammatory fibroblasts and myofibroblasts in pancreatic cancer (10.1084/jem.20162024). **B** Schematic overview of co-culture system. Credit: Sheng et al., TAK1 promotes an immunosuppressive tumor microenvironment through cancer-associated fibroblast phenotypic conversion in pancreatic ductal adenocarcinoma (10.1158/1078-0432.CCR-24-1004). **C** Direct contact co-culture experimental strategies. Credit: Zhang et al., Cancer-associated fibroblasts nurture LGR5 marked liver tumor-initiating cells and promote their tumor formation, growth, and metastasis (10.1002/cam4.6408). **D** Spontaneous reorganization of cancer cells and CAFs into macroscopic mini-tumors. Credit: Strating et al., Co-cultures of colon cancer cells and cancer-associated fibroblasts recapitulate the aggressive features of mesenchymal-like colon cancer (10.3389/fimmu.2023.1053920). **E** Schematic overview of co-culture system. Credit: Schuth et al., Patient-specific modeling of stroma-mediated chemoresistance of pancreatic cancer using a three-dimensional organoid-fibroblast co-culture system (10.1186/s13046-022-02519-7). **F** Illustration of a 3D co-culture experiment and results of immunofluorescence staining. Credit: Feldmann et al., Mesenchymal Plasticity Regulated by Prrx1 Drives Aggressive Pancreatic Cancer Biology (10.1053/j.gastro.2020.09.010). **G** Schematic diagram of a mixed inverted suspension culture model. Credit: Xu et al., A novel CAF-cancer cell crosstalk-related gene prognostic index based on machine learning: prognostic significance and prediction of therapeutic response in head and neck squamous cell carcinoma (10.1186/s12967-024-05447-6). All the above pictures are used under CC BY 4.0 (https://creativecommons.org/licenses/by/4.0/).

In early conventional culture of organoids, transwell system is used in order to explore the influence of paracrine factors where CAFs are inoculated in top chamber and tumor organoid in bottom chamber, or vice versa [19]. Recently, matrigel-submerged organoid culture system composed of cancer organoids and CAFs is used to investigate the interaction between them (Fig. 1A–C), in the system tumor cells are obtained from patient-derived samples or established tumor cell lines [20–22] and CAFs are typically derived from primary fibroblasts isolated from tumor stroma or alternatively generated by activating normal fibroblasts [23].

In most co-culture organoid models, tumor cells are firstly inoculated into matrigel and CAFs are added when organoids reach a certain diameter [24, 25] (Fig. 1D). In other experiments, primary tumor organoids derived from surgically resected cancer tissues are inoculated in a suitable medium, then fibroblasts and organoid forming units are mixed and planted in a co-culture matrix containing collagen or matrigel [24, 26–28] (Fig. 1E). In few experiments, CAFs are first inoculated on a 24-well plate overnight, then organoids composed of tumor cells are mixed with matrigel and placed on top of CAFs [29], for example, a pancreatic ductal adenocarcinoma (PDAC) organoid is established as described above to investigate whether mesenchymal plasticity of fibroblasts is modulated by genetic depletion of Prrx1 [30] (Fig. 1F).

Although traditional matrigel-submerged organoid cultures system has several advantages, such as, high throughput, efficiency, easy operation and retention of tumor heterogeneity; heterogeneous source of matrigel introduces xenogeneic components which may interfere with studies involving human cells and restrict clinical translation [31, 32], batch-to-batch inconsistencies in matrigel and other animal-derived matrices further complicate reproducibility [31].

### Mixed and inverted suspension culture

Mixed and inverted suspension culture is a special culture method, cell mixture composed of cancer cells and CAFs at different ratio is prepared and cell suspension droplets are added on a dish lid, the lid is turned over and the cells are cultured for 3 days to form cell spheroids which are inoculated into matrigel subsequently [33] (Fig. 1G). Suspending droplets under inner surface of plate lid reduce contamination risks from debris or exogenous factors. However, challenges persist, technical complexity arises from the need for precise gel droplet handling and plate inversion, which can introduce variability in large-scale applications.

### Three-dimensional (3D) microfluidic co-culture system

**Fig. 2:**
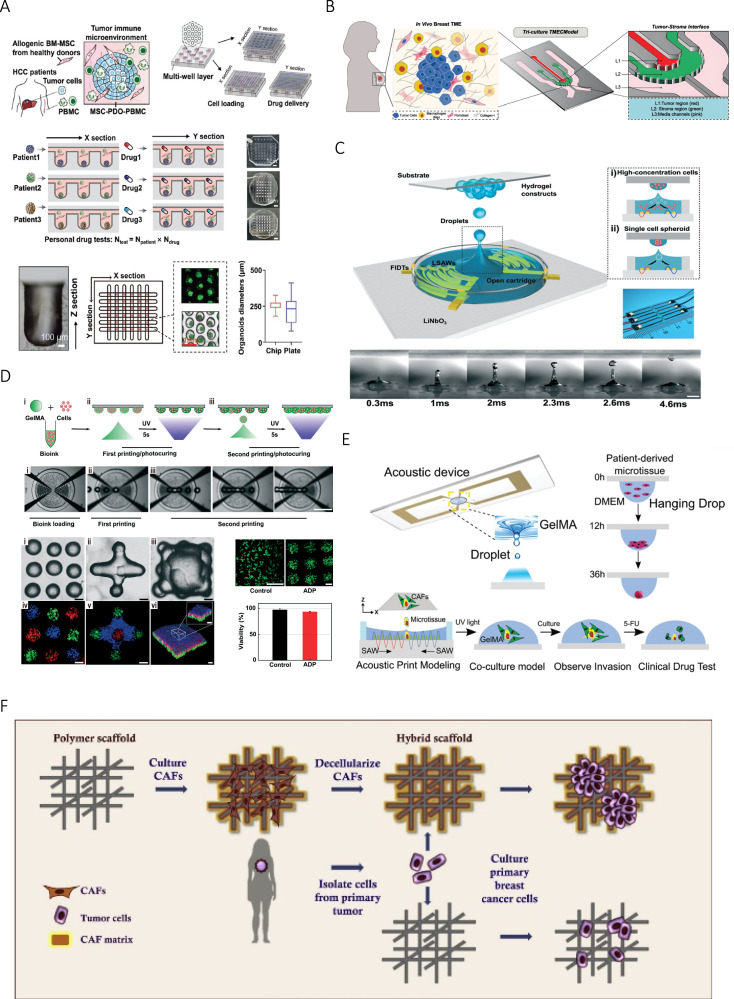
Special cultivation methods of organoids. **A** Scheme of a microfluidic chip for high-throughput organoid culture. Credit: Zou et al., Micro-Engineered Organoid-on-a-Chip Based on Mesenchymal Stromal Cells to Predict Immunotherapy Responses of HCC Patients (10.1002/advs.202302640), CC BY 4.0 (https://creativecommons.org/licenses/by/4.0/). **B** Scheme of a human organotypic microfluidic tumor. Credit: Ravi et al., Tumor Microenvironment On-A-Chip and Single-Cell Analysis Reveal Synergistic Stromal-Immune Crosstalk on Breast Cancer Progression (10.1002/advs.202413457), CC BY 4.0 (https://creativecommons.org/licenses/by/4.0/). **C**, **D** Schematic illustration of ADP and 3D hydrogel construct. Used with permission of Royal Society of Chemistry, from The acoustic droplet printing of functional tumor microenvironments, Chen et al., 21, 1594334-1; permission conveyed through Copyright Clearance Center, Inc. **E** Scheme of acoustic bioprinting for organoid. Used with permission of Royal Society of Chemistry, from Modeling cancer metastasis using acoustically bio-printed patient-derived 3D tumor microtissues, Chen et al., 10, 1594343-1; permission conveyed through Copyright Clearance Center, Inc. **F** Tissue-mimetic 3D scaffold for breast tumor-derived organoid. Reprinted from Colloids and Surfaces B: Biointerfaces, 180, Nayak et al., Tissue Mimetic 3D Scaffold for Breast Tumor-derived Organoid Culture Toward Personalized Chemotherapy, 334-343, 2025, with permission from Elsevier.

As conventional culture methods face difficulties in large-scale organoid culture and high-throughput drug screening, microfluidic culture method emerges. This micro-engineered organoid chip uses designed micropores and channels to ameliorate tumor engineering model and improve predictive accuracy for immunotherapy response evaluation [34–36]. The controllable shape and size of organoids can be realized by control of liquid flow rate and design of microfluidic channel, real-time imaging could also be achieved [34]. In an experiment, microfluidic chips composed of microarrays with different geometric shapes have been established, the top and middle layers of microarrays are devised for delivering drug and loading cells, the bottom layer for organoid culture and drug test [34] (Fig. 2A). In another study, a 3D microfluidic co-culture equipment has been established as an organotypic tumor model which consists of parallel tumor and stromal regions similar to structure of early TME [37] (Fig. 2B).

In microfluidic tumor models, spatial architecture recapitulates critical molecular mechanisms of tumor-stromal crosstalk, facilitating drug target discovery while enabling imaging-based quantification for proliferation and migration. Microfluidic systems also have limitations, for example, this model is limited by high-throughput production and short of a real 3D environment [38], and increased gel volume and cell demand due to expanded platform, exacerbated cell loss and complicated perfusion dynamics restrict its application [39].

### Acoustic droplet printing (ADP)

The above cultivation methods have their own shortcomings, hydrogel embedding lacks spatial control over stromal-tumor interactions, hanging drop method suffers from low throughput and inconsistent spheroid sizes, and organoids-on-chips are complex and costly. By comparison, ADP overcomes these disadvantages via high cell viability and precise spatial patterning, enabling scalable and functional CAFs-tumor co-culture system.

In previous study, acoustic printer comprised of a lithium niobate substrate, an annular chamber and a pair of focused interdigital transducers is established [40] (Fig. 2C, D). A similar acoustic 3D printing equipment composed of a program control part, a precision control receiving platform and an observation platform has been built by Hui Chen [17], the droplets are excited onto receiving plate which moves in accordance with the programmed coordinates to realize accurate printing (Fig. 2E). Patient-derived tumor organoids are acoustically printed using hanging-drop culture surrounded by CAFs in Gelatin Methacryloyl (GelMA) to form a CAFs-tumor spheroid, high concentration of cells achieves faster establishment of size-controlled organoids within a week, which is applicable for rare samples [41]. In another research, an acoustic bioprinting device is used to organize CRC and healthy organoids into 3D architecture precisely to mimic the diseased colorectum of patients [42].

While acoustic bioprinting can create basic 3D structures, fabricating intricate vascular systems remains challenging. Acoustic forces during printing can induce shear stress and cavitation effects, potentially compromising cell viability [42–44]. Current acoustic bioinks often lack optimal mechanical properties and biocompatibility, limiting the ability to support long-term organoid maturation [43, 44]. Therefore, adjusting ink formulations to balance viscosity, cell adhesion, and acoustic responsiveness remains a critical hurdle.

### 3D mechano-mimetic scaffold

The use of organoid-based models to recapitulate complex structure remains challenging, which could be resolved through bridging cell biology with scaffold engineering. Scaffolds composed of natural materials or synthetic polymers furnish structural underprop to guide cell proliferation, adhesion and differentiation. Advanced manufacturing techniques, such as 3D printing, enable accurate control on porosity, mechanical properties, and biomimetic architecture, mimicking ECM features which is critical for organoid maturation [45, 46]. A study describes a method for culturing breast cancer-derived organoids using scaffold which offers advantages including CAF-derived ECM and enhances cell adhesion and cell viability to capture patient-specific drug responses [47] (Fig. 2F).

Decellularized ECM scaffolds derived from liver or thymus retain native biochemical cues and promote vascular network formation [48]. Natural materials, while having excellent biocompatibility, lack mechanical stability and may degrade unpredictably, compromising long-term culture integrity. A high-throughput screening model has been established in 96-well plates with synthetic polycaprolactone scaffolds, HeLa cells and CAFs are cocultured within the scaffolds to evaluate drug response [49]. Despite advancements in scaffold design, scaffold rigidity often deviates from physiological mechanical cues, impairing cell differentiation and function [50], and challenges persist in recapitulating native tissue complexity; future researches need to prioritize dynamical response, biomimetic structure, bioactivity and functional integration.

## Regulations of CAFs on tumor cells

The important role of CAFs in cancer development is emphasized by their impacts on multiple features of cancer, such as cell proliferation, invasion, metastasis, ECM remodeling, immune suppression, angiogenesis and therapeutic responses. CAFs can modulate cancer cells by releasing chemokines and cytokines, at the same time, cancer cells generate specific molecules to modify phenotype of CAFs, resulting in a positive feedback loop to promote tumor development further [51].

### CAFs participate in ECM remodeling

The dense fibrous matrix in tumor is composed of various ECM components, including collagen, fibronectin and laminin. CAFs play an important part in cell rearrangement and structural formation, contributing to organization of tumor stroma and establishment of TME [20]. Pancreatic cancer organoids composed of cancer cells and isolated CAFs display incremental expressions of vimentin and fibronectin [27]. Pancreatic stellate cells (PSCs) conditioned by BRCA-deficient cancer cells reveal attenuated collagen production activity [23]. In addition, CAFs can also remodel ECM via secretion of matrix metalloproteinases (MMPs) that degrade ECM components [3].

### CAFs regulate proliferation of tumor cells

**Fig. 3:**
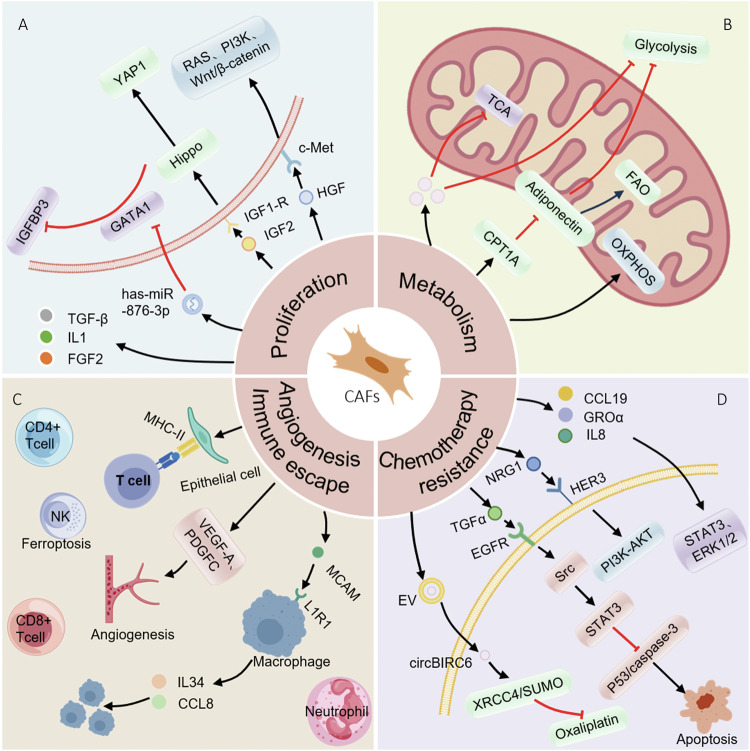
CAFs modulate proliferation, metabolism, angiogenesis, immune microenvironment and chemotherapy resistance of tumors. **A** CAF-released cytokines, such as IGF2 and HGF, combine with their receptors and active Hippo-YAP1, RAS and Wnt/β-catenin signaling pathways, driving tumor proliferation. EVs secreted by CAFs are rich in miR-876-3p, enhancing survival and proliferation. **B** Overexpressed CPT1A in CAFs downregulates secretion of adiponectin, shifting metabolism from glycolysis to FAO in CAFs and sparing glucose for cancer cells. **C** CAFs secrete chemokine, leading to an immunosuppressive state when binding their receptors. CAFs secrete VEGF-A and PDGF-C, promoting angiogenesis. **D** CAF-secreted TGF-α activates EGFR/Src/STAT3 survival pathway to inhibit chemotherapy-induced apoptosis by downregulating p53/caspase-3. CAFs facilitate antiandrogen resistance in prostate cancer via NRG1/HER3 axis.

CAFs have long been considered as protumorigenic components in TME due to their involvement in desmoplasia and secretion of factors that promote cancer cell proliferation and survival [52–54] (Fig. 3A).

CAFs enable cancer cells proliferation via a series of complicated signaling networks. CAFs drive proliferation via secreting GROα, CCL19 and IL-8 that activate STAT3 and ERK1/2 pathways in hormone receptor-positive breast cancer [55]. CAFs promote CRC progression by secreting insulin-like growth factor 2 (IGF2) which subsequently binds to IGF1R on cancer cells, activating PI3K/AKT and Hippo/yes-associated protein 1 (YAP1) signaling pathways, driving tumor proliferation [56]. CD142^high^ fibroblasts promote CRC cell proliferation by secretion of hepatocyte growth factor (HGF) which binds to c-Met receptor on CRC cells, triggering downstream RAS, Wnt/β-catenin and PI3K pathways to drive cell proliferation [57, 58]. Other mechanisms also involve CAF-promoted proliferation, CAFs secrete extracellular vehicles (EVs) enriched with hsa-miR-876-3p which is transferred to tumor cells to enhance cell survival and proliferation in OSCC cells [59].

### CAFs facilitate cancer invasion and metastasis

Conditioned media from EP3-deficient CAFs promotes epithelial-to-mesenchymal transition (EMT) in MMTV-PyMT mammary tumor organoids in vivo, indicating that CAFs contribute to tumor progression through paracrine mechanisms [60]. In a CRC organoid, CAFs promote cancer invasion by secreting IGF2 which binds to IGF1R on CRC cells, activating downstream Hippo-YAP1 signaling [56]. CCL2, MMP2 and vascular endothelial growth factor-A (VEGF-A) secreted by CAFs drive EMT and angiogenesis, facilitating colon cancer peritoneal metastasis [61]. CAFs derived from brain metastases of breast cancer secrete CXCL12 and CXCL16 to promote brain metastases [62].

**Fig. 4:**
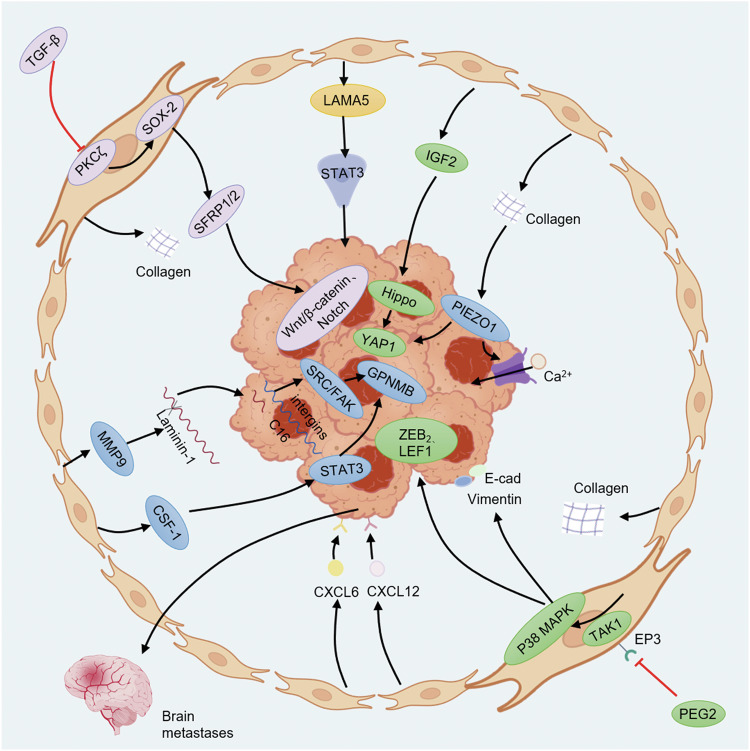
CAFs regulate tumor invasion, initiation and metastasis. Decrease of PKCζ in stroma caused by TGF-β leads to upregulation of SOX2 in CAFs, driving formation of SFRP1/2 CAFs, enhancing migration and invasion via Notch and Wnt/β-catenin signaling. CAFs promote initiation of pancreatic cancer through LAMA5/ITGA4/STAT3 signaling. CAFs secrete CXCL12 and CXCL16 which lead to brain metastasis of breast cancer. CAFs secrete collagen which activate mechanical sensor PIEZO1 in cancer, triggering YAP1 signaling and promoting metastasis.

CAFs increase migratory and invasive potential by upregulating genes or pathways involved in ECM remodeling and EMT [63]. CAF-secreted MMP9 cleaves laminin-111 to bioactive peptides which bind to integrins and trigger invasion in breast cancer organoid [64]. In a CAF co-culture system, CAFs in diffuse-type gastric cancer promote tumor EMT by secreting fibulin-5 which activates cAMP response element-binding protein pathway to enhance cell migration and invasion [65]. Stiffened TME caused by CAF-secreted ECM activates mechanosensor PIEZO1 in GC, triggering calcium influx and YAP1 signaling to promote metastasis [66]. Overall, CAFs exert a significant impact on tumor cell invasion and metastasis (Fig. 4), understanding these mechanisms is essential for developing effective therapeutic strategies.

### CAFs modulate metabolism of tumor cells

The metabolically dependent relationship between CAFs and cancer cells is concerned with cell survival of each other (Fig. 3B). In KRAS-mutant CRC, CAFs reprogram metabolism by upregulating glycolysis, inhibiting tricarboxylic acid cycle, and decoupling oxidative and non-oxidative arms of pentose phosphate pathway, thereby enhancing cell proliferation and survival through increased lactate secretion and glutamine utilization [67]. In PDAC, CAFs reduce secretions of alanine and aspartate by promoting glutamine-dependent reductive carboxylation, promoting a metabolic niche where PDAC cells rely on oxidative phosphorylation (OXPHOS) [68].

How tumor cell-intrinsic alterations impact on CAFs to indulge metabolic demands of tumorigenesis has been of great interest. CAFs in peritoneal metastatic CRC upregulate carnitine palmitoyltransferase 1 A, shifting metabolism from glycolysis to fatty acid oxidation (FAO), this metabolic symbiosis-CAFs oxidize fatty acids to spare glucose for cancer cells [61]. In pancreatic tumors, SETD2-deficient tumor cells activate adipogenic pathway of CAFs through BMP2-BMPR signaling axis, promoting lipid synthesis; this type of lipid-rich CAFs supplies lipids to tumor cells through ABCA8a transporter in turn, promoting FAO-OXPHOS metabolic remodeling and stemness enhancement [69].

### CAFs trigger cancer initiation and regulate tumor stemness

CAFs facilitate tumor initiation and progression, foster cell plasticity and stemness via dynamic interactions with tumor. CAFs induce transdifferentiation of pancreatic acinar cells into duct cells through LAMA5/ITGA4/STAT3 signaling and promote initiation of pancreatic cancer [70]. CAFs enhance growth of LGR5^+^ cells and organoid initiation through both paracrine signaling and direct contact in liver cancer, specific knockout of LGR5 expressing cells suppresses CAF-mediated tumor formation, growth, and metastasis in vivo [20].

When specific signaling pathways are activated by corresponding stimuli from cancer cells or TME, CAFs achieve a phenotype to maintain cancer stemness. In a CRC organoid established from mice with simultaneous mutations of Trp53, Apc, Tgfbr2 and Kras, stromal loss of PKCζ induced by TGF-β reprograms colonic fibroblasts into pro-tumorigenic CAFs which promote stemness of epithelium through PKCζ-SOX2-SFRP1/2 signaling axis [71]. Colonic mesenchymal stromal cells are found to have CAF-like properties in high fat diet-fed mice and accelerate expressions of cancer stem cell-related markers in colon organoids via production of Wnt2b [72]. In OSCC, CAF-derived lactate enhances cancer stemness through inhibiting ubiquitination degradation of macrophage stimulating 1 [73]. CAF-derived CXCL12 activates downstream CXCR4 signaling, contributing to tumor stemness in prostate cancer organoid [51].

### The role of CAFs on immunomicroenvironment

CAFs inhibit recruitment and activation of immune cells, creating a favorable environment for tumor immune escape, subgroups with high CAFs-cancer cell crosstalk-related gene prognostic index have lower immune scores [33]. Poor efficacy of immune-checkpoint blockade therapy in prostate cancer is attributed to high collagen deposition and limited immune cell infiltration mediated by CAFs [74]. CAFs in CRC secrete chemokine CXCL12 which recruits specific immune cell subsets and leads to an immunosuppressive state when binding to CXCR4 or CXCR7 [75]. CAFs induce epithelial cells to express MHC-II molecules and inflammatory signatures like IFN-γ response, modulating immune interactions in PDAC [76].

CAFs and immune cells are essential components of TME, their interaction constitutes a major player for tumor immune suppressive microenvironment. PKCζ-deficient CAFs and senescent CAFs create an immunosuppressive microenvironment by limiting CD8^+^ T cell infiltration, increasing neutrophil recruitment and downregulating interferon pathways [71, 77]. Prrx1-proficient CAFs may foster an immunosuppressive microenvironment with decreased infiltration of CD8⁺ and CD4⁺ T cells [30]. Recent advances indicate that the immunosuppressive function of CAFs also attribute to the interactions between CAFs and other immune cells. In a GC organoid model, CAFs upregulate expressions of ferroportin 1 and hephaestin, causing overload of iron in NK cells; CAFs also induced ferritinophagy via upregulating expression of nuclear receptor coactivator 4. Ferroptosis of NK cells fosters a permissive environment for tumor cells [78]. In CRC, Lepr-lineage CAF-expressed CD146 interacts with IL-1 receptor, leading to secretion of IL-34/CCL8 to recruit tumor-associated macrophages and an immunosuppressive niche [79].

CAFs are a highly heterogeneous population, some specific CAFs contribute to cancer inhibition by reshaping immune microenvironment. In a murine GC model, antigen-presenting CAFs (apCAFs) exhibit stronger tumor-killing ability by driving functional differentiation of CD4⁺ T cells to an activated state [74]. In a pancreatic cancer model, interferon-response CAFs (ifCAFs) secrete cytokines that directly promote shift of tumor-associated neutrophils (TANs) from protumorigenic TAN1 to antitumor TAN2 [80].

Clarifying diversity of CAF subtypes and the regulatory mechanism will contribute to identification of potential therapeutic methods by taking advantage of the distinct activities of CAFs (Fig. 3C). However, due to inconvenience in obtaining sufficient immune cells, short survival time of immune cells in vitro and hardship to choose suitable culture medium for different cells, it is difficult to establish co-culture organoid models composed of tumor cells, CAFs and immune cells which lead to limited research in this field using co-culture organoids.

### CAFs modulate angiogenesis

CAFs support significantly tumor growth through facilitating angiogenesis (Fig. 3C). In a context of OSCC, CAFs secrete VEGF-A and platelet-derived growth factor-C (PDGF-C), directly stimulating endothelial cell sprouting and lumen formation, CAFs contribute to ECM remodeling by producing matrisomes which are essential for neovascularization [81]. Overexpression of nicotinamide N-methyltransferase in CAFs reduces histone methylation at VEGF-A promoter, enhancing chromatin accessibility and promoting transcription factor JUNB binding, thereby upregulating VEGF-A expression [81].

### CAFs account for chemotherapy resistance

The diameters of liver tumor organoids treated with sorafenib, regorafenib or 5-fluorouracil increase significantly when co-cultured with CAFs, suggesting that CAFs lead to resistance to chemotherapy [19]. In pancreatic cancer organoids, CAF-secreted collagen decreases exposure of cancer cells to gemcitabine [27]. Overexpression of CXCR4 in CAFs is associated with bortezomib resistance in cholangiocarcinoma [82]. In bladder cancer, CAFs promote chemoresistance to cisplatin by augmenting DNA repair gene ERCC4 through CXCL14/CCR7/STAT3 axis, reducing cisplatin-induced DNA damage and apoptosis [83].

Recently, the role of CAFs in resistance to molecular targeted agents has been given increasing attention. CAFs confer resistance to EGFR inhibitors via RTK/RAS pathway which can be reversed by c-Met inhibition in colorectal cancer organoid [26]. CAFs facilitate antiandrogen resistance in prostate cancer via NRG1/HER3 axis which triggers PI3K/AKT signaling, sustaining cell survival and proliferation [84]. CAFs isolated from hormone receptor-positive breast cancer secrete GROα and CCL19 which reduce efficacy of fulvestrant via activating STAT3 and ERK1/2 signaling in patient-derived organoids [55].

CAFs drive upregulation of EMT-related genes, such as COL1A1 and vimentin, and EMT is associated with reduced chemotherapy sensitivity [85–87]. In esophageal adenocarcinoma, IL-6 secreted by CAFs activates STAT3 pathway and leads to enhanced treatment resistance to carboplatin and paclitaxel via promoting EMT [88]. In CRC organoids, CAF-secreted HGF activates RTK/RAS signaling in tumor cells, bypassing EGFR inhibition [26]. EMT mediated by combination of HGF and FGF2 secreted by CAFs to their receptors abrogates chemoresistance of PDAC organoid to gemcitabine, 5-fluorouracil and paclitaxe [28].

CAF-secreted EVs also involves drug resistance, stable circular RNA circBIRC6 in CAFs is transmitted to pancreatic cancer cells via EVs, which promote SUMOylation of XRCC4 at lysine 115, facilitating XRCC4 chromatin localization and augmenting DNA repair efficiency, increasing survival under oxaliplatin treatment [89]. Pro-tumorigenic CAFs-secreted EVs enriched with miR-876-3p which targets GATA-binding protein 1 promote cisplatin resistance in OSCC [59]. Altogether, resistance to cancer treatment is a major clinical obstacle, robust evidence has indicated that CAFs are involved in anticancer therapeutic resistance (Fig. 3D).

In summary, CAFs exhibit multifaceted roles in TME in primary and metastatic tumors, influence behavior of cancer cells profoundly and promote cancer progression and development through extensive interactions with cancer cells and other stromal cells (Table 2).

**Table 2:** The factors secreted by CAFs and their regulation on tumor cells.

Modulations of CAFs on tumor cells	Cytokine/ Chemokine/ Protein
Remodeling ECM	Collagen, laminin, fibronectin, ZEB-1, vimentin [27], MMPs [23]
Regulating proliferation	IL-1 and TGFβ [94], FGF2 [53], GROα, CCL19, IL-8 [55], IGF2 [56], EVs with hsa-miR-876-3p [59], HGF [57], IL-6 [88]
Affecting invasion and metastasis	LAMA5 [70], IGF2 [56], CCL2 [61], VEGF-A [61], MMP2 [61], VEGF-C [91], collagen [107], SOX2 [71], TGF-β, IL-6 and VEGF [20], HGF [30], CCL5 and SOX9 [60], CXCL12 and CXCL16 [62], CSF-1 and MMPs [64]
Affecting tumor metabolism	adiponectin [61]
Accounting for chemotherapy resistance	Collagen [27], CXCR4 [82], NRG1 [84], TGFα [29], CCL19 [55], HGF [26], CD44-HGF interactions [28], IL-6 [88], EVs [89], EVs enriched with miR-876-3p [59], HGF [30]
Regulating tumor stemness	CXCR4 [51], HGF [108]
Modulating immunomicroenvironment	CXCL9/10/11[25], MCAM [79], IL-8 [55], ITGB1[76]
Modulating angiogenesis	VEGFA and PDGFC [81]

## Modulation of tumor cells on CAFs

### Cancer cells activate CAFs

**Fig. 5:**
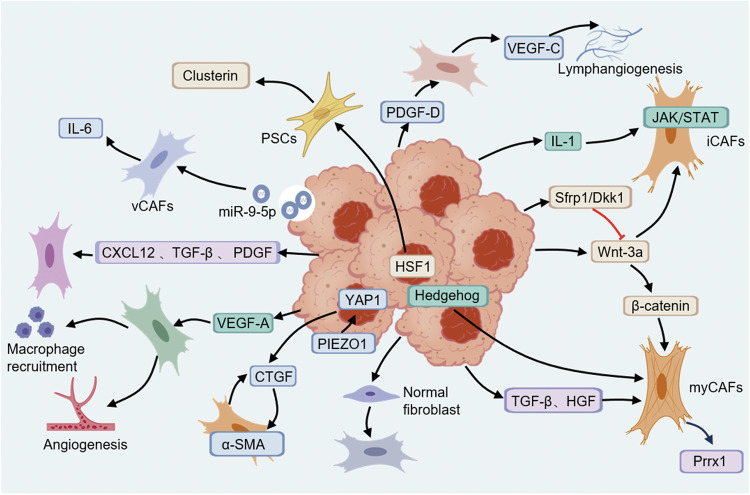
Modulation mechanisms of tumor cells on CAFs. PIEZO1-YAP1 signaling in cancer cells stimulates infiltration of CAFs to form a stiffened matrix. Tumor cell-derived exosomes initiate production of IL-6 in vCAFs. Tumor-secreted Wnt drives differentiation of myCAFs via β-catenin signaling. Tumor cells secrete HGF and TGF-β which upregulate Prrx1 in CAFs, driving their transition to myCAFs.

Based on fibroblast-cancer cell co-culture organoids, it is observed that activation of CAFs is directly or indirectly modulated by cancer cells (Fig. 5). Some studies have indicated that activation of CAFs by cancer cells is mediated by secretory components related to ECM remodeling [90]. In matrix gel infiltrated by cancer cells, a great deal of activated fibroblasts gather at the front edge of cancer cells [33].

In CRC, CAFs are reprogrammed into contractile, immunosuppressive cells through tumor-derived TGF-β and RTK/RAS signaling pathways [26]. Activation of PIEZO1/YAP1 axis in GC cells stimulates CAFs infiltration to form a stiffened microenvironment [66]. PDGF-D secreted from cholangiocarcinoma promotes VEGF-C secretion from CAFs and enhances lymphangiogenesis and lymph node metastasis [91]. BRCA-mutated pancreatic cancer cells reprogram PSCs into clusterin^+^ CAFs via heat shock factor 1 signaling [23]. Furthermore, in cholangiocarcinoma, tumor cell-derived exosomal miR-9-5p initiates production of IL-6 in vCAFs to accelerate tumor proliferation [92]. Exosomes containing TGF-β secreted by bladder cancer cells lead to activation of SMAD signaling which transforms normal fibroblasts to CAFs [93].

### Cancer cells modulate shift of CAFs

TGF-β and IL-1/JAK/STAT signaling pathways are major regulatory pathways for formation of myCAFs and iCAFs, TGF-β secreted by PDAC cells acts on neighboring myCAFs, inhibiting induction of iCAF phenotype; while IL-1 acts on distant CAFs, activating JAK/STAT signaling pathway and maintaining phenotype of iCAFs [94]. TGF-β secreted by PDAC cells binds to TGFBR2 on PSCs and initiates PSCs differentiation into myCAFs, TGF-β further promotes upregulation and secretion of amphiregulin (EGFR ligand) in myCAFs via autocrine, sustained activation of EGFR/ERBB2 maintains the myCAF phenotype and suppresses transition to iCAFs [8]. TGF-β and HGF produced by tumor cells upregulate Prrx1 expression in CAFs, driving transition to myCAF-like states characterized by α-SMA expression, collagen deposition and HGF secretion [30]. In CRC model, tumor-secreted Wnt activates β-catenin signaling in CAFs, driving differentiation of myCAFs, while Wnt antagonists promote a shift toward iCAFs [63]. In Scribble-deficient mouse PDAC organoid, downregulated IL-1 impairs activation of PSCs into iCAFs; additionally, Hedgehog signaling pathway, which is important for activation of myCAFs, is downregulated in Scribble-null tumors [95].

## Therapies targeting interactions between tumor cells and CAFs

In solid tumors, complicated interacting net between CAFs and cancer cells in TME creates a supportive niche for tumor progression and metastasis. These interactions underscore therapeutic potential of targeting CAFs-tumor cell communication. Numerous preclinical trials have confirmed some potential targets for CAFs, manifesting prominent efficacy of some CAF-targeted treatments; herein, a great deal of findings on potential targeting strategies for CAFs are summarized.

### Targeted therapies for special CAFs

The heterogeneity of CAFs addresses the need for more refined characterization to enhance therapeutic efficiency. By analyzing BRCA-mutant pancreatic cancer, an increase of immunoregulatory clusterin^+^ CAF subset is found to be mediated by heat shock factor 1, and inhibition of heat shock factor 1 with aglaroxin C reverses clusterin induction in CAFs [23]. Lepr⁺ pericryptal stromal cells differentiate into MCAM⁺ CAFs during colorectal carcinogenesis, inhibition of differentiation of Lepr-lineage cells could disrupt formation of MCAM⁺ CAFs, thereby reducing recruitment of tumor-associated macrophages [79]. Previous study has identified CD142^high^ CAFs as a key promoter of CRC progression, and inhibitor of heat shock protein 90 reduces CD142^high^ CAFs through negative selection, disrupting their supportive niche [57]. In intrahepatic cholangiocarcinoma, targeting vCAFs or inhibiting IL-6/IL-6R axis in tumor cells can be an effective way to reduce degree of malignancy [92]. However, heterogeneity of CAFs and absence of particular lineage markers make it difficult to target them precisely without interfering normal tissues, highlighting that more precise treatment strategies are requisite.

### Therapies targeting shift of CAFs

An extremely essential aspect towards a pro-cancerous TME is the conversion of stromal fibroblasts to CAFs, inhibition of CAF transformation provides an actionable therapeutic intervention. In PDAC, CAFs shorted of TGF-β1-activated kinase 1 undergo phenotypic conversion to myCAFs, characterized by α-SMA upregulation and reduced inflammatory cytokines; inhibition of TGF-β1-activated kinase 1 decreases collagen deposition and enhances CD8^+^ T-cell infiltration [21]. In CAFs-integrated pancreatic cancer organoids, all-trans retinoic acid restores mechanical quiescence of PSCs and restrains activity of CAFs, reducing productions of vimentin and fibronectin [27]. In another research, STING agonists promote ifCAF phenotype in vitro and in vivo, transformed ifCAFs reveal antitumor activities by directly modulating polarization of TAN [80].

Transformation of highly heterogeneous CAFs is mediated by intricate signaling and CAF shift can be inhibited by blocking these signaling pathways. YAP1 mediates plastic transform of CAFs to protumorigenic roles, and inhibition of YAP1 induces a phenotypic switch to Lym-CAFs, reducing ECM deposition and promoting immune cell infiltration [25]. STAT3 inhibitors, such as silibinin, increase myCAF/iCAF ratio and improve treatment outcomes for pancreatic cancer [96]. Neratinib, an EGFR/ERBB2 inhibitor, preferentially depletes CD90⁻ myCAFs and alters myCAF/non-myCAF ratio in tumors [8].

### Therapies targeting crosstalk between CAFs and tumor cells

Due to the contribution of CAFs to therapy resistance, blocking paracrine factors between CAFs and tumor cells attenuates the function of CAFs. In CRC organoids, CAFs promote cancer invasion by secreting IGF2 which binds to IGF1R on CRC cells, activating downstream Hippo-YAP1 signaling, combinatorial treatment with IGF1R inhibitor and YAP1 inhibitor demonstrates enhanced antitumor effects [56]. CAFs promote invasion by inducing expression of glycoprotein nonmetastatic B in breast cancer cells, and silence of glycoprotein nonmetastatic B blunts the influence of CAFs on cancer cells [64]. In head and neck squamous cell carcinoma, chemoresistant CAFs upregulate TGF-αwhich binds to EGFR on cancer cells, activating EGFR/Src/STAT3 survival pathway and repressing chemotherapy-induced apoptosis; anti-EGFR antibody restores chemosensitivity in murine models co-planted with rCAFs and cancer cells [29]. CAFs derived from brain metastases of breast cancer secrete high levels of CXCL12 and CXCL16, blocking CXCR4 (receptor of CXCL12) on tumor cells using an antagonist or neutralizing CXCL16 reduces metastasis [62]. These findings may constitute potential therapeutic strategies for chemoresistant by disrupting communication between CAFs and cancer cells.

### Targeting metabolic symbiosis between CAFs and cancer cells

Cancer cells remould nearby wound-healing cells to CAFs to maintain their metabolism, highlighting therapeutic potential of disrupting metabolic symbiosis between CAFs and cancer cells. Hypoxia reprograms metabolism of CAFs to favor glycolysis and lactate production, supporting iCAFs activation and stabilization; targeting HIF-1α reduces iCAFs-mediated inflammation and angiogenesis [97]. CAFs upregulate carnitine palmitoyltransferase 1 A, a rate-limiting enzyme for FAO, in peritoneal metastatic CRC, and inhibiting carnitine palmitoyltransferase 1 A using etomoxir or inhibition of glycolysis reduces survival of CRC cells [61]. Metformin disrupts OXPHOS in PDAC microtumors co-cultured with CAFs, reducing their redox state and metabolic plasticity. By targeting CAFs-mediated metabolic reprogramming, metformin sensitizes PDAC cells to oxaliplatin and photodynamic therapy [68]. PYCR1, a key enzyme in proline synthesis, is highly expressed in tumor stroma and CAFs, lowering PYCR1 in CAFs decreases production of tumor collagen and inhibits tumor proliferation and metastasis [98].

### Targeting CAFs using biological therapies

In pancreatic cancer organoids, a pH-sensitive nanometer system with heat shock protein 47 transforms activated PSCs into static-state cells [99]. Follistatin like protein 1 derived from CAFs abets ferroptosis NK cells, a novel treatment combined application of deferoxamine and neutralizing antibody against follistatin like protein 1 significantly reduces CAFs-triggered ferroptosis of NK cells and enhances cytotoxicity of NK cells to GC cells [78]. The advent of chimeric antigen receptor (CAR) T cell therapy ushers cancer therapy to a new paradigm of personalized medicine. Highly expressed ULBP2 activates TGF-β signaling pathway to drive fibroblast-to-CAFs transformation; ULBP2 CAR-T cells specifically recognize and eliminate ULBP2-expressing cells via their anti-ULBP2 scFv, suppressing activation of CAFs and collagen deposition [100]. CD70 has been identified as a target of CAFs and tumor cells in PDAC and CRC patients, CD70-targeted CAR NK cells stimulated by IL-15 effectively eliminate CD70^+^ CAFs and tumor cells, improving survival rate of mice carrying CD70^+^ tumors [101].

Based on insights of CAF-derived EVs in tumor progress, EVs from CAFs carrying anti-miR-876-3p effectively deliver inhibitor to tumor cells to sensitize OSCC cells to cisplatin by suppressing pro-survival IGF-1R signaling [59]. Significant upregulation of circBIRC6 in CAFs-derived EVs is definitely correlated with chemoresistance to oxaliplatin, antisense oligonucleotide inhibitor targeting circBIRC6 leads to increased DNA damage and apoptosis in pancreatic cancer cells and significantly reduces chemotherapy resistance by disrupting XRCC4/SUMOylation axis [89].

### Invalid treatment targeting CAFs

Although the strategy of targeting CAFs holds great prospects; increased risk of metastasis and worsening prognosis associated with CAF depletion strategy in relevant mouse models and clinical trials reveal a subtle balance between tumor support and tumor suppression functions of CAFs [102]. Depleting α-SMA⁺ myofibroblasts in pancreatic cancer mouse model induces immunosuppression, accelerates tumor progression and reduces survival, highlighting failure of such CAFs-targeted strategies [103, 104]. In another mouse PDAC, inhibition of hedgehog signaling reduces proliferation of stromal connective tissue, but accelerates tumor growth and progression, providing a potential explanation for the disappointing clinical trial results using standard chemotherapy and hedgehog pathway inhibitors [105].

Here, we review the function of CAFs with a focus on how CAF subsets contribute to therapeutic resistance in cancer. We also recapitulate current breakthroughs in targeting CAFs to overcome anticancer therapeutic resistance and summarize emerging CAFs-targeted modalities (Table 3).

**Table 3:** Therapies targeting CAFs or interactions between tumor cells and CAFs.

Function	Method or drug candidate	Target	Mechanism	Reference
Reverse chemotherapy resistance/Increase chemotherapy sensitivity	Plerixafor	CXCR4	Reversing bortezomib resistance by reducing CAFs and increasing CD8⁺ T-cell infiltration	[82]
	Antisense oligonucleotide	circBIRC6	Increasing DNA repair and chemotherapy resistance via XRCC4/SUMOylation axis	[89]
	CAF-P EVs carrying anti-miR-876-3p	miR-876-3p/GATA1/IGFBP3 axis	Overcoming cisplatin resistance in OSCC	[59]
	Cetuximab	EGFR	Inhibiting CAFs-mediated activation of EGFR/Src/STAT3	[29]
	Metformin	Mitochondrial complex I	Sensitizing cancer cells to oxaliplatin and photodynamic therapy by disrupting OXPHOS	[68]
Inhibit tumor invasion and metastasis	Picropodophyllin and verteporfin	IGF1R and YAP1	IGF2 derived from CAFs binds to IGF1R and activates Hippo-YAP1 signaling	[56]
	Neratinib	EGFR/ERBB2	Preferentially depleting CD90⁻ myCAFs and altering myCAF/non-myCAF ratio	[8]
	shRNA	GPNMB	GPNMB promoting breast cancer cell invasion	[64]
	Plerixafor or antibody for CXCL16	CXCR4 or CXCL16	Secretion CXCL12 and CXCL16 by CAFs derived from brain metastases	[62]
	Etomoxir	CPT1A	Disrupting CAFs metabolic reprogramming	[61]
	siRNAs	PYCR1	Lower PYCR1 in CAFs decrease production of collagen	[98]
Affect the tumor immune microenvironment	ABT-199	Senescent CAFs	Suppressing senescent CAFs in PDAC contributing to immunosuppression	[77]
	Deferoxamine and FSTL1 antibody	Fe^2+^, FSTL1	Deferoxamine reducing CAF-induced NK cell ferroptosis, FSTL1 antibody reducing fibronectin in CAFs	[78]
	Neutralizing MCAM antibodies	MCAM	Reducing TAM recruitment and tumor-promoting inflammation	[79]
	Aglaroxin C	HSF1	HSF1 augments clusterin^+^ CAFs, remodeling ECM	[23]
	HIF-1α single-guide RNAs	HIF-1α and LDHA	Reducing iCAF-mediated inflammation	[97]
	5Z-7-oxozeaenol	TAK1	5Z-7-oxozeaenol decreases collagen deposition, enhances CD8^+^ T-cell infiltration	[21]
Affect CAFs	BCLi and HSP90i	CRC cells and CAFS	BCLi induces apoptosis of CAFs, HSP90 inhibitor reduces CD142^high^ CAFs	[57]
	CD70-CAR-IL-15 NK cells	CD70^+^ CAFs	CD70-directed CAR NK cells eliminates CD70^+^ CAFs	[78]
	siRNA	YAP1	YAP1 transforms CAFs from protumorigenic to immunosupportive roles	[25]
	Silibinin	STAT3	Reducing iCAF activity, increase myCAF/iCAF ratio	[109]
	STING agonists	ifCAFs	STING agonists promote ifCAF phenotype	[80]

## Prospect in the research about CAFs and tumor cells

Although CAFs have long been investigated as a crucial player in cancer development and therefore represent an attractive therapeutic target, many clinical trials targeting CAFs have ended in failure, and in some cases, promoted cancer development, highlighting that sophisticated identities and functions of CAFs far exceeds the current cognition. Consequently, analyzing the relationship between heterogeneity and multiple pro-and anti-tumorigenic functions of CAFs in a context-dependent mode has important significance.
